# Increased RHAMM expression relates to ovarian cancer progression

**DOI:** 10.1186/s13048-017-0360-1

**Published:** 2017-09-27

**Authors:** Stephanie T. Buttermore, Mitchel S. Hoffman, Ambuj Kumar, Anne Champeaux, Santo V. Nicosia, Patricia A. Kruk

**Affiliations:** 1 Department of Pathology & Cell Biology, University of South Florida, Morsani College of Medicine, 12901 Bruce B. Downs Blvd., MDC 11, Tampa, FL 33612 USA; 2 Gynecologic Oncology, Moffitt Cancer Center, Tampa, FL 33612 USA; 3 Department of Internal Medicine, University of South Florida, Tampa, FL 33612 USA; 4 Department of Obstetrics & Gynecology, University of South Florida, Tampa, FL 33612 USA

**Keywords:** Hyaluronan-mediated motility receptor, Ovarian cancer, Immunohistochemistry

## Abstract

**Background:**

Elevated hyaluronan-mediated motility receptor (RHAMM) has been reported to contribute to disease progression, aggressive phenotype and poor prognosis in multiple cancer types, however, RHAMM’s role in ovarian cancer (OC) has not been elucidated. Therefore, we sought to evaluate the role for RHAMM in epithelial OC.

**Results:**

Despite little to no expression in normal ovarian surface epithelium, western immunoblotting, immunohistochemical staining and enzyme linked immunosorbent assay showed elevated RHAMM levels in clinical tissue sections, omental metastasis and urine specimens of serous OC patients, as well as in cell lysates. We also found that RHAMM levels increase with increasing grade and stage in serous OC tissues and that RHAMM localizes to the apical cell surface and inclusion cysts. Apical localization of RHAMM suggested protein secretion which was validated by detection of significantly elevated urinary RHAMM levels (*p* < 0.0001) in OC patients (116.66 pg/mL) compared with normal controls (8.16 pg/mL). Likewise, urinary RHAMM levels decreased following cytoreductive surgery in OC patients suggesting the source of urinary RHAMM from tumor tissue. Lastly, we validated RHAMM levels in OC cell lysate and found at least 12× greater levels compared to normal ovarian surface epithelial cells.

**Conclusion:**

This pilot study shows, for the first time, that RHAMM may contribute to OC disease and could potentially be used as a prognostic marker.

## Background

Commonly described as the “silent killer”, ovarian cancer (OC) is the fifth leading cause of cancer related deaths in women and it has the highest mortality of all gynecological malignancies [1]. Roughly one in 70 women will develop OC in her lifetime with only a 45% 5 year survival [2]. When disease has undergone metastatic spread, the survival rate drastically decreases from > 90% in early stage disease to less than 30% in late stage [1]. OC lethality is largely due to ambiguous symptoms, emergence of drug resistance, disease reoccurrence and lack of reliable screening methods all leading to late stage diagnosis. This underscores the need to improve our understanding of this disease and its etiology. Delineating key players driving disease would help elucidate potential molecular targets for treatment as well as for monitoring and detecting disease.

Receptor for hyaluronan-mediated motility (RHAMM) belongs to a group of hyaladherins, which share a common ability to bind to hyaluronan (HA). Based on subcellular localization, RHAMM performs multiple functions. Intracellularly, RHAMM is involved in microtubule spindle assembly, thereby contributing to cell cycle progression [3]. On the extracellular surface, RHAMM forms a trimeric complex with cluster differentiation 44 (CD44) and HA to activate cell signaling pathways that promote migration, invasion and cell proliferation [4]. While RHAMM is overexpressed in hematological malignancies and solid tumors arising from prostate [5], bladder [6] and breast [7], it is not known whether RHAMM contributes to OC. Although minimally expressed in normal tissue, elevated RHAMM in breast cancer (BC) and colorectal cancer (CRC) is associated with poor clinical outcome and more a aggressive cancer phenotype [7, 8]. Herein, we sought to determine if RHAMM similarly contributes to OC progression.

## Methods

### Clinical specimens

With University of South Florida Institutional Review Board approval (studies #Pro00003119 & 4739) and patient consent, tissues were collected from a cohort of women who had undergone primary surgery with complete surgical staging for epithelial ovarian cancer (EOC) or low malignant potential (LMP) tumors as defined by as benign but still containing abnormal cells, at the Moffitt Cancer Center and the University of South Florida (Table 1). This gynecologic oncology database was also used to select women who had undergone oophrectomy due to cystadenoma or had their ovaries removed for unrelated pathology. All tissue specimens were fixed with 10% formalin, paraffin-embedded, sectioned and stained with hematoxylin and eosin (H & E). The slides were reviewed by pathologists (AC, SVN) to confirm histologic diagnosis according to the International Federation of Gynecology and Obstetrics (FIGO) classification system.

**Table 1:** Summary of the patient cohort

	*N* = 33 Patients	LGSC	HGSC
Normal	5		
Serous OC	22	7	15
Stage			
I	2	2	0
II	3	0	3
III	9	3	6
IV	8	2	6
Other			
Normal Fallopian Tube	6	N/A	N/A
Breast Cancer	2	1	1

University of South Florida Institutional Review Board approval and patient consent was obtained for prospective (studies #Pro00003119, #Pro00000903) and retrospective (study #106004) collection of urine samples. Annonymized urine samples from healthy controls (*N* = 29), patients with benign gynecological pathology (*N* = 32) or OC (*N* = 150) were released for research from the Moffitt Cancer Center and the University of South Florida. All samples were centrifuged at 3000 x g and the supernatant was aliquoted and frozen at −20 °C before analyses were conducted.

### Immunohistochemistry

For immunohistochemical (IHC) studies, formalin-fixed paraffin sections were cut at 3 μm and dried overnight at room temperature (RT) then deparaffinized and rehydrated. Sections were incubated in BLOXALL™ Blocking Solution (Vector Laboratories, Burlingame, Ca) for 20 min to block endogenous peroxidase activity. Antigen retrieval was achieved by placing slides in 1× solution Antigen Unmasking Solution (Vector Laboratories, Burlingame, Ca) brought to a boil and maintained at 95 °C for 30 min on a hot plate. Specimens were then immunostained using rabbit anti-human CD168 (Ca#:PA5–32309 ThermoFisher Scientific, Waltham, MA) primary antibody (1:100) for 1 h and Vectastain® Elite ABC Kit (Vector Laboratories)**,** Dako’s EnVision™ and HRP Rabbit (DAB+) kit according to the manufacturer’s instructions, then counterstained with modified Mayer’s haematoxylin, dehydrated through graded alcohol, cleared with xylene, and mounted with resinous mounting medium. To control variability, all samples were stained at the same time and with the same lot of reagents. BC was used as an internal positive control while negative controls were obtained by substitution of primary antibody with normal rabbit serum.

### Evaluation of RHAMM staining

A minimum of 100 cells were counted at a final magnification of 400X per tissue section. Immunostaining of RHAMM was scored based on average percent of positive epithelial cells (0, negative or no staining; 1 +, < 30%; 2 +, 30–50% and; 3 +, > 50%) and on staining intensity (negative, weak, moderate and strong). Cellular localization of RHAMM was also assessed as cytoplasmic, membranous, nuclear or stromal.

### Images

Images were acquired on a digital *Olympus DP-20* camera under a *Leica dmire2* microscope. *Olympus* micro imaging software *CellSens* platform was used to acquire and process images. Images were taken with a final magnification of 100 × and 400 ×.

### Western blot (WB)

For urinary analysis, normal and OC urine samples of equivalent volume were centrifuged at 16,000 x g using 30,000 kDa microfilters (Millipore, Bedford, MA) to concentrate the urine specimens. Concentrated urine samples were subjected to a Bradford protein assay and 30μg of protein were electrophoresed via 10% sodium dodecyl sulfate-polyacrylamide gel electrophoresis (SDS-PAGE) and transferred to nitrocellulose membrane. Membranes were blocked for 1 h at RT in 5% milk in tris-buffer saline with tween. Membranes were incubated overnight at 4 °C in monoclonal rabbit anti-CD168 RHAMM antibody (Ca# ab124729 abcam®, Cambridge, MA) and then incubated for 1 h at RT in goat anti-rabbit HRP conjugated antibody (Thermo Fisher Scientific, Waltham, MA). Protein bands were visualized using SuperSignal West Femto Substrate (Thermo Fisher Scientific, Waltham, MA) followed by densitometric analysis using *Image Studio Lite Version 5.0* software program.

For analysis of RHAMM levels in cultured cells, the SV 40-Large T-Ag-transfected human OSE (HIOSE-118 and HIOSE-121) and OC (OVCAR5, OV90, and SKOV3) cells were cultured in Medium 199/MCDB 105 (Sigma, St. Louis, MO) with 5% fetal bovine serum and gentamicin. All cells were incubated at 37 °C with 5% CO_2_. Cells were washed in PBS, trypsinized, pelleted, and washed 1–2 times in cold PBS. Cells were lysed in CHAPS buffer and 30 μg of protein was separated via 10% sodium dodecyl sulfate polyacrylamide gel electrophoresis (SDS-PAGE). Proteins were transferred to polyvinylidene fluoride (PVDF) membranes and blocked in 5% milk in Tween 20-Tris buffered saline. Blots were incubated in their respective primary antibodies overnight, followed by incubation with a horseradish peroxidase-(HRP-) conjugated secondary antibody (Fisher, Pittsburgh, PA), and developed via enhanced chemiluminescence substrate (ECL) (Pierce/Fisher, Pittsburgh, PA) followed by densitometric analysis using *Image Studio Lite Version 5.0* software program. Antibodies used were monoclonal rabbit anti-CD168 RHAMM antibody (Ca# ab124729 abcam®, Cambridge, MA) previously validated by Coulson-Thomas et al. doi:10.1074/jbc.M114.557447 [9].

### Human HMMR/CD168/ RHAMM sandwich enzyme-linked immunosorbent assay (ELISA)

RHAMM ELISA (LifeSpan BioSciences, Inc., Seattle, WA) was performed according to the manufacturer’s recommended instructions for urine sample specimens. Prior to performing the experiments, all samples were thawed to RT and centrifuged to remove particulate matter. Plates were read using a microplate reader (BioTek ELx800, Winooski, VT) with a 450 nm wavelength filter.

### Statistical analysis

Samples for RHAMM ELISA were run in duplicate and concentrations calculated as per manufacturer’s protocol. Data were subjected to the student T-Test for determination of statistical differences. *p* ≤ 0.05 was considered statistically significant.

## Results

### RHAMM is overexpressed in OC

Immunological staining was performed on 41 tissue sections from 33 women. The sample population provided sections of normal ovarian surface epithelium (OSE) (*n* = 5), serous OC (*n* = 22), omental metastasis (*n* = 7), lymph node metastasis (*n* = 1), and normal fallopian tube (FT) (*n* = 6). Though these samples comprise a small pilot study, they are representative of a typical clinical practice with regards to histological distribution (Table 1). BC was used as a positive control (*n* = 2).

**Fig. 1:**
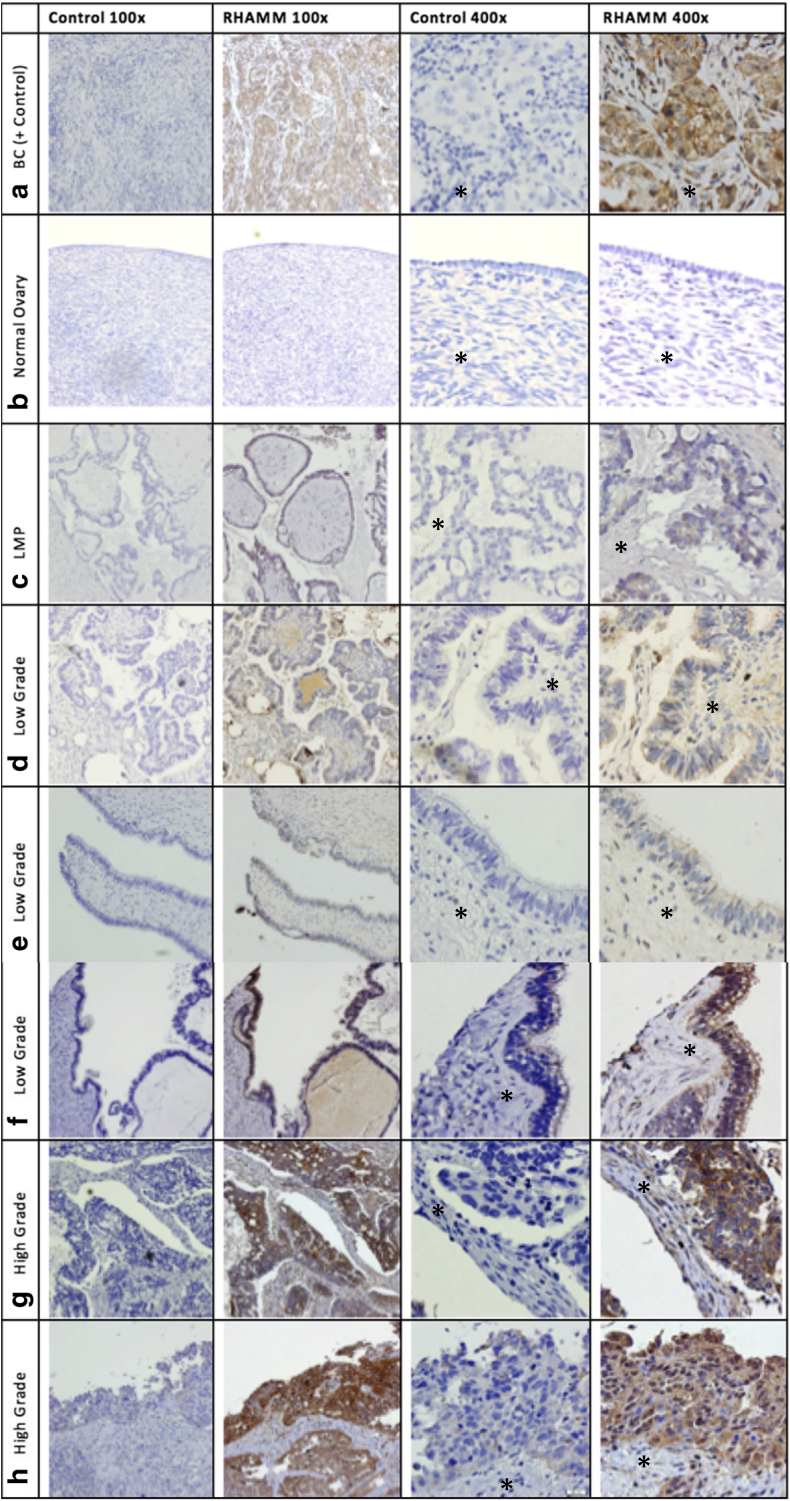
RHAMM staining is elevated in OC tissue specimens. Representative photographs of IHC staining for RHAMM [anti-CD168 polyclonal antibody (PA5–32309) with a 1:100 dilution] in (**b**). normal, (**c**). LMP (**d**, **e**, **f**). LGSC and (**g**, **h**). HGSC. High grade BC was used as a positive control (**a**). Control sections were incubated with non-immune serum. *Asterisks* indicate stroma*. Original magnification 100× and 400×

Overall, we found that 91% (20/22) of serous OC stained positively for RHAMM with levels of staining intensity ranging from weak (< 30% from each field, *N* = 4), moderate (30–50%, *N* = 7) to strong (> 50%, *N* = 9) while 0% (0/5) of normal OSE stained for RHAMM. (Fig. 1, Table 2) Staining patterns, as depicted by intense, punctate/diffuse cytoplasmic staining, seen in BC positive control were consistent with previous reports of RHAMM in BC where intense staining is predominately in the cytoplasm and nucleus, but negative in the stroma [10, 11].

**Table 2:** Quantification of RHAMM IHC

			% Staining			Staining intensity		
	N	Positive	< 30%	30–50%	> 50%	Weak	Moderate	Strong
Normal	5	0						
Serous OC	22	20/22	4/22	7/22	9/22	5/22	6/22	9/22
Grade								
Low	7	6/7	2/7	3/7	1/7	3/7	2/7	1/7
High	15	14/15	2/15	4/15	8/15	2/15	4/15	8/15
Stage								
I	2	2/2	1/2	1/2	0	1/2	1/2	0
II	3	3/3	1/3	1/3	1/3	0/3	2/3	1/3
III	9	8/9	1/9	3/9	4/9	3/9	1/9	4/9
IV	8	7/8	1/8	2/8	4/8	1/8	2/8	4/8
Fallopian Tube	6	6/6	0	1/6	5/6	1/6	2/6	3/6

RHAMM expression was predominately localized in the cytoplasm in 91% (20/22) of the serous OC specimens while the surrounding stromal tissue remained negative (Fig. 1, Table 3). Further, membranous staining was seen in 27% (6/22) of serous OC (Fig. 1d, e, f &h) and 18% (4/22) of serous OC specimens demonstrated nuclear staining (Fig. 1h). Low-grade serous carcinoma (LGSC) displayed membranous RHAMM staining localized to the apical cell surface (Fig. 1d, e, f & h). Interestingly, we noted RHAMM staining in OC cysts (Fig. 1d&f).

**Table 3:** Subcellular localization of RHAMM

Localization	N	Positive	Cytoplasm	Membrane	Nuclear	Stroma
Normal	5	0				
Serous OC	22	20/22	20/22	6/22	4/22	1/22
Grade						
Low	7	6/7	6/7	1/7	1/7	1/7
High	15	14/15	14/15	5/15	3/15	0
Stage						
I	2	2/2	2/2	0	0	0
II	3	3/3	3/3	1/3	1/3	0
III	9	8/9	8/9	3/9	2/9	0
IV	8	7/8	7/8	2/8	1/8	1/8
Fallopian Tube	6	6/6	5/6	5/6	2/6	2/6

### RHAMM intensity in primary serous OC tumors increases with grade and stage

High-grade serous carcinoma (HGSC) sections displayed intense punctate staining, LGSC displayed mostly weak, diffuse staining while LMP sections had almost no staining (Fig. 1, Table 2). Although percentages of positive staining was similar in LGSC (6/7 or 86%) and HGSC (14/15 or 93%) serous OC, increased staining intensity correlated with increasing grade. Strong RHAMM staining intensity was calculated as 14% and 53% of LGSC versus HGSC specimens respectively (Fig. 1, Table 2). Additionally, we found a tendency for RHAMM staining intensity to increase with stage. Strong RHAMM staining intensity levels were shown to be 0%, 33%, 44% and 50% of total stage 1, 2, 3 and 4 specimens respectively (Table 2).

**Fig. 2:**
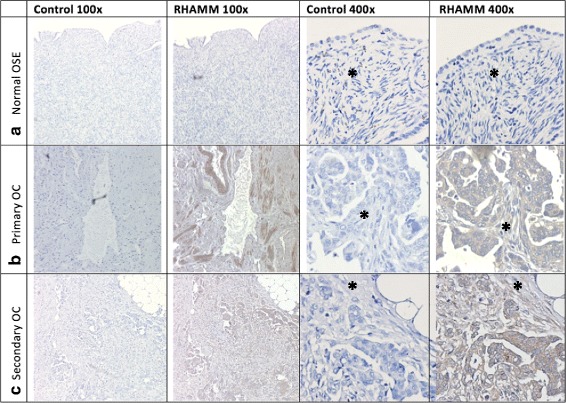
Primary and metastatic OC stain equally for RHAMM. Representative photographs of IHC staining for RHAMM in multiple sections from within the same patient of (**a**) normal, (**b**) primary serous adenocarcinoma and (**c**) secondary omental metastatic tissue. Control sections were incubated with non-immune serum. *Asterisks* indicate stroma*. Original magnification 100× and 400×

Incidentally, we also noted RHAMM staining in primary tumors and their respective metastases for eight serous OC patients. We found positive RHAMM staining in 88% (7/8) of the primary tumors and 88% (7/8) of their omental or 100% (1/1) of lymph node metastases. Similar RHAMM staining percentages, intensities and cellular localization were seen in primary tumors and their respective metastases among all 8 patient samples (Fig. 2, Table 4).

**Table 4:** RHAMM immunostaining patterns shown in primary and metastatic tumors

Patient	Description	Grade	Staining	% Staining	Intensity
P1	Normal Ovary	–	–	0	Negative
P1	Primary OC	low	+	1	Weak
P1	Omental metastasis	low	+	1	Weak
P2	Primary OC	low	+	2	Weak
P2	Omental metastasis	low	+	2	Weak
P3	Normal Ovary	–	–	0	Negative
P3	Primary OC	high	+	3	Strong
P3	Omental metastasis	high	+	3	Moderate
P4	Primary OC	high	+	1	Weak
P4	Omental metastasis	high	+	1	Weak
P5	Primary OC	high	–	0	Negative
P5	Omental metastasis	high	–	0	Negative
P6	Primary OC	high	+	3	Strong
P6	Omental metastasis	high	+	3	Strong
P7	Primary OC	high	+	2	Moderate
P7	Omental metastasis	high	+	2	Moderate
P8	Primary OC	high	+	2	Moderate
P8	Lymph node metastasis	high	+	1	Weak

### Normal fallopian tube epithelium (FTE) stains intensely for RHAMM

**Fig. 3:**
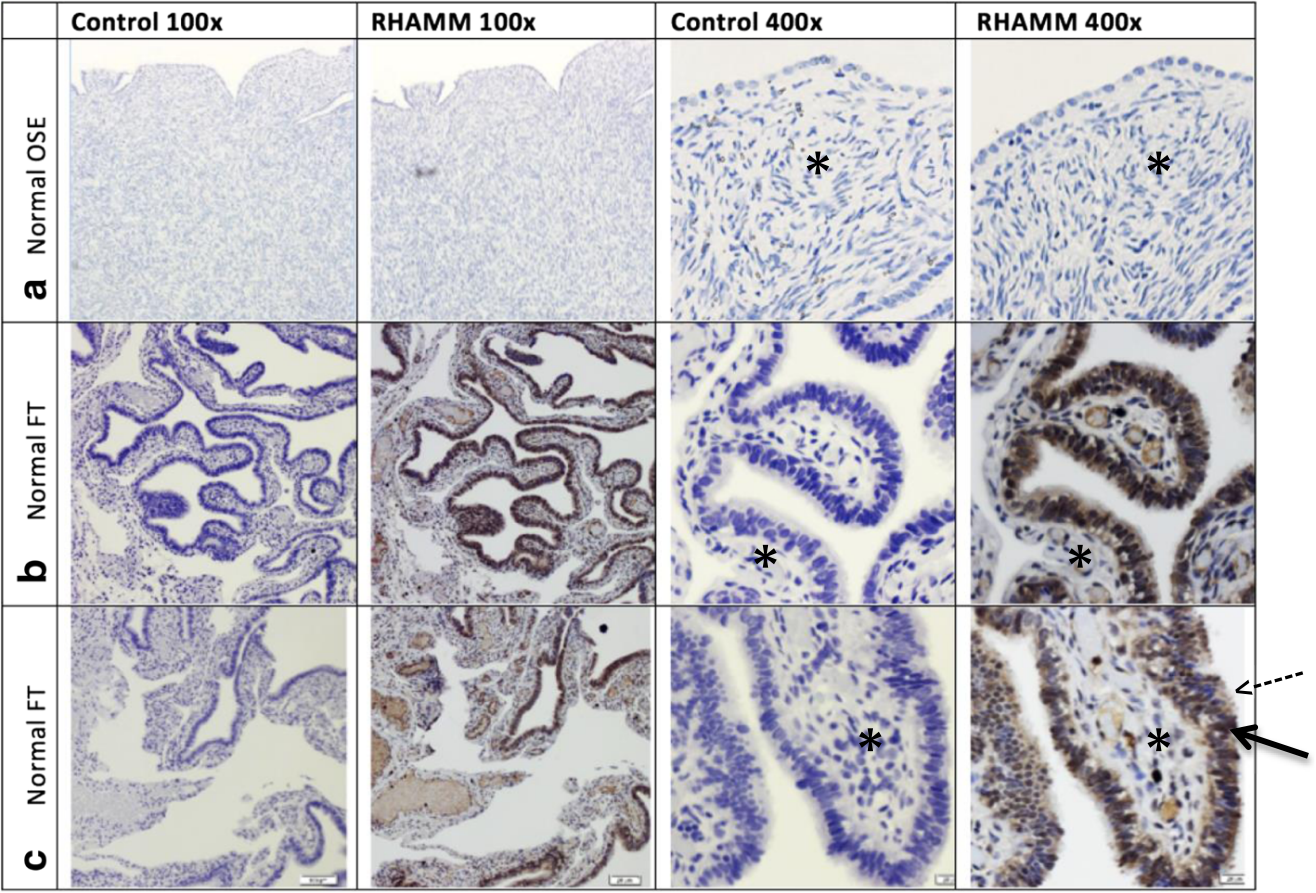
Normal FTE stains intensely for RHAMM. Representative photographs of IHC staining for RHAMM in normal (**a**). OSE and (**b**, **c**). FT. Control sections were incubated with non-immune serum. *Asterisks* indicate stroma* and *arrows* indicate secretory (*solid*) and ciliated (*dotted*) FTE cells. Original magnification 100× and 400×

Compared to normal OSE, which failed to stain for RHAMM, we found 100% (6/6) positive RHAMM staining in all normal FTE specimens examined (Fig. 3). There was moderate to strong staining limited to the surface of the fimbrial epithelium, cytoplasm and nucleus while stromal elements (Fig. 3 asterisks) did not stain for RHAMM. Specifically, there was strong staining in the nucleus and apical surface of secretory cells (Fig. 3, solid arrows) while ciliated epithelial cells (Fig. 3, dotted arrow) showed moderate apical and cytoplasmic staining but negative nuclear staining (Fig. 3).

### Urinary RHAMM levels are elevated in OC patients

**Fig. 4:**
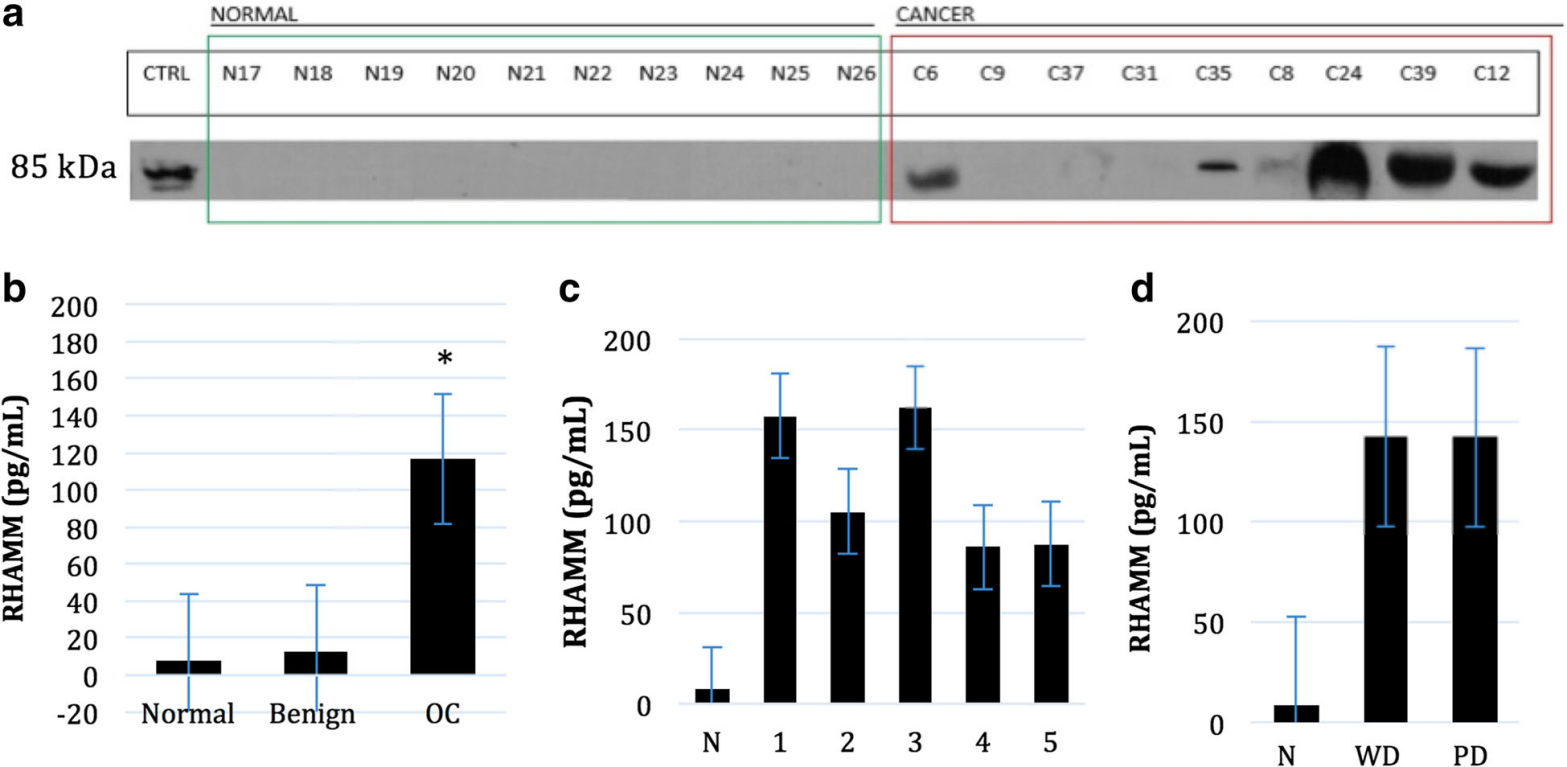
Urinary RHAMM levels are elevated in OC patients. **a** Concentrated urine samples of equivalent volumes from normal controls (*N* = 10) and patients with serous OC (*N* = 9) were screened for RHAMM by WB. Membranes were incubated with anti- RHAMM (1:1000) overnight and visualized with enhanced chemiluminescent. BC cell lysate (MCF-7) was used for positive control. **b** Urinary samples were examined by ELISA for RHAMM levels in normal (*N* = 29), OC (*N* = 150) and benign gynecological disease (*N* = 32), according to disease stage (**c**) and (**d**) grade. Results are expressed as a mean pg/mL RHAMM ± SE and represented as a histogram where *p* ≤ 0.05 was considered statistically significant

Since RHAMM expression appeared localized to cytoplasm, cell surface membrane and especially at the apical cell surface in HGSC, normal FT and potentially within ovarian cystic fluids (Fig. 1), we sought to determine whether RHAMM could be secreted by OC cells and, thereby, be detected in bodily fluids. Urinary analysis of RHAMM protein levels from OC patients measured by WB indicated the presence of RHAMM protein in 6/9 (66.7%) urine samples from patients with serous OC, while RHAMM was undetectable in all (10/10) urine samples from healthy controls (Fig. 4a). For more quantitative analyses, ELISA studies revealed urinary RHAMM levels from serous OC (*N* = 150) averaged almost 15X higher than normal controls (*N* = 29) (Fig. 4b, Table 5). Urinary levels of RHAMM in normal controls averaged 8.16 pg/mL, compared to 116.66 pg/mL in OC patient urine (*p* < 0.0001). Additionally, ELISA measurements of urinary RHAMM from 32 women with benign gynecological diseases including ovarian cysts, uterine fibroids and teratomas averaged 12.47 pg/mL, slightly higher than normal controls, but still significantly lower than OC RHAMM levels (p < 0.0001) (Fig. 4b). Lastly, average urinary RHAMM levels were not significantly different between disease stage (Fig. 4c) or grade (Fig. 4d).

**Table 5:** Clinical parameters of urinary patient cohort

Sample	N	Average urinary RHAMM (pg/mL)	Range lower limit	Range upper limit	*p*-value
Normal	29	8.16433368	−8.50288	54.43993895	
Benign	32	12.4764694	−1.029525763	85.95183057	*p* < 0.0001
OC	150	116.662456	−27.1929	1702.52388	*p* < 0.0001
STAGE					*P* = 0.249
1	11	157.635708	14.12994	424.42521	
2	4	105.348346	37.508515	143.22621	
3	45	162.35244	−10.77318	1702.52388	
4	6	85.8344317	12.838485	175.459295	
5	11	87.5899564	22.47945	239.703255	
GRADE					*P* = 0.280
LGSC	23	142.265278	14.12994	424.42521	
HGSC	27	141.993346	−10.77318	1702.52388	

*Values are expressed as an average urinary RHAMM level (pg/mL) where *p* < 0.05 was considered statistically significant

### Urinary RHAMM levels decrease after cytoreductive surgery in OC

**Fig. 5:**
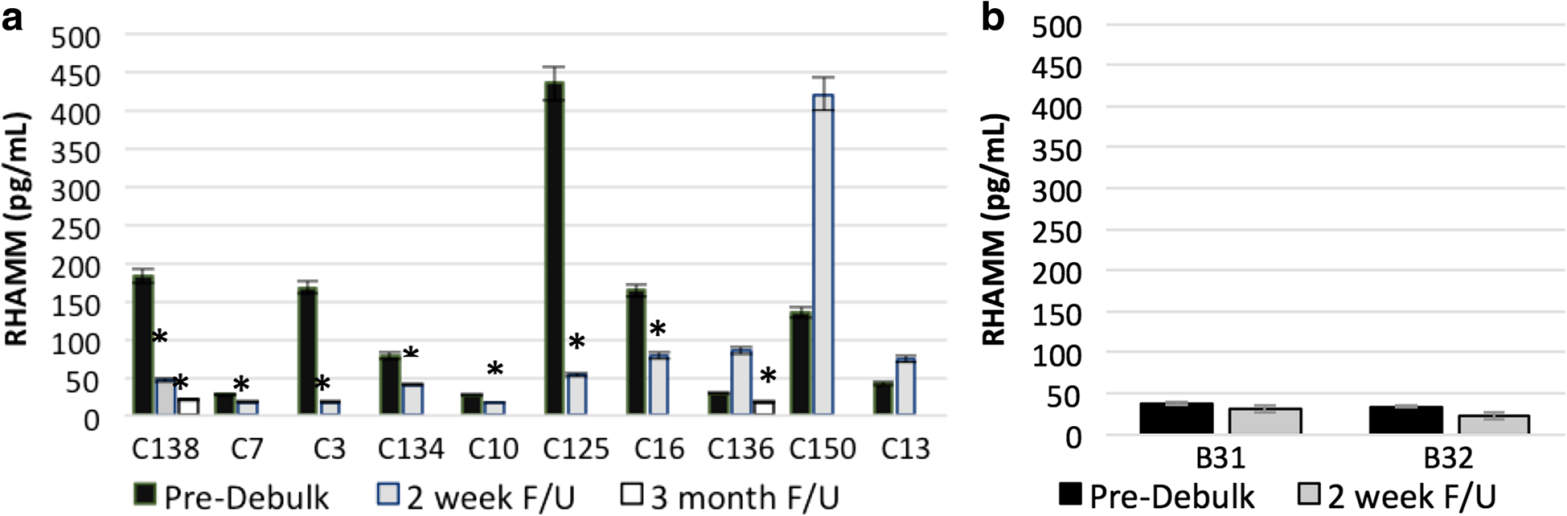
Urinary RHAMM levels decrease after tumor debulking. Urinary levels of RHAMM were measured by ELISA from (**a**) 10 OC patients and (**b**) 2 patients with benign LMP ovarian tumors prior to tumor removal (*black bars*), within 2 weeks of debulking surgery (*gray bars*) and 3 months post debulking surgery (*white bars*) where available. Results are expressed as a mean pg/mL RHAMM ± SE and represented as a histogram where *p* ≤ 0.05 was considered statistically significant

Urinary RHAMM levels were also measured by ELISA in 10 OC patients (Fig. 5a) and two patients with LMP ovarian tumors (Fig. 5b) immediately prior to initial cytoreductive surgery, within 2 weeks of cytoreductive surgery and, where possible, at a t3 month post-operative follow-up. We found up to 89% reduction in urinary RHAMM levels post-cytoreductive surgery compared to pre-cytoreductive surgery in 70% (7/10) of OC patients 2 weeks post-surgery and in 80% (8/10) of OC patients 3 months post-surgery. An increase in urinary RHAMM levels 2 weeks post-cytoreductive surgery was noted in 30% (3/10) OC patients compared to pre- cytoreductive surgery urinary RHAMM levels. In contrast, urinary RHAMM measurements in patients with benign disease pre- and post-tumor reduction remained low (average 36 pg/mL), thereby remaining relatively unchanged with surgical intervention.

### RHAMM levels are elevated in OC cell lines

**Fig. 6:**
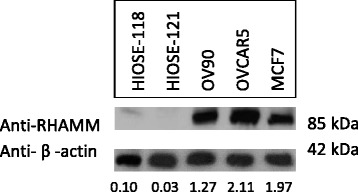
RHAMM is overexpressed in cultured OC cells. Protein levels of RHAMM were measured by WB in HIOSE-118, HIOSE-121, OV90 and OVCAR5 cells. WB was performed on protein cell lysates using monoclonal rabbit anti-CD168 RHAMM antibody at a 1:1000 dilution. Densitometric values were quantified using image analysis (Image Studio Lite Version 5.0) of target protein bands to β-actin levels where MCF-7 cells were used as a positive control

Validation of RHAMM expression in OC was performed measuring protein levels by WB of cellular RHAMM in OC and HIOSE cell cultures. RHAMM levels were consistently elevated as a single band at 85 kDa in OC cell lines (OV90 and OVCAR5) compared to IOSE cells (HIOSE-118 and HIOSE-121) and with BC cell line (MCF7) as a positive control (Fig. 6). Densitometric values confirm about 40× and 12× more RHAMM in OV90 cells compared to normal OSE and about 70× and 20× more RHAMM in OVCAR5 cells compared to control measured by Image Studio Lite computer software program.

## Discussion

Overall survival of OC has not improved for several years due to poor understanding of its pathogenesis, late diagnosis, emergence of drug resistance and lack of reliable biomarkers. Consequently, in order to better elucidate the etiology of this disease, the aim of this pilot study was to determine if, like other cancer types, RHAMM is overexpressed in OC and whether RHAMM could, likewise, promote OC progression.

We show for the first time that RHAMM expression is elevated in serous OC compared to normal OSE. We observed 91% of serous OC patients demonstrated positive RHAMM staining which was localized primarily to the cytoplasm, cell membrane, cystic fluid and, occasionally, the nucleus. RHAMM staining appeared to be specific to epithelial tumor cells since the stroma failed to stain. We also showed that RHAMM staining intensity increased with increasing cancer grade and showed a tendency to increase with stage. Levels of RHAMM appear to be dependent upon the extent of disease progression where LMP showed negligible to no staining, LGSC and early stage specimens demonstrated weak punctate staining while HGSC and late stage specimens typically demonstrated intense, but rather diffuse RHAMM staining. High levels of RHAMM in aggressive colorectal cancer tumor budding cells are associated with higher grade, poor survival, increased lymphatic invasion and nodal metastasis [12]. Additionally, invasive BC cell lines express higher levels of RHAMM [13] and IHC staining in 189 mammary carcinomas revealed that elevated RHAMM in lobular carcinomas is correlated with more invasive behavior and reduced overall patient survival time [11]. Therefore, increasing levels of RHAMM seen in OC could contribute to an invasive and progressive phenotype.

Since recent studies suggest that HGSC arises from the FTE [14], we also subjected normal FTE to IHC staining for RHAMM. Distinct molecular markers of HGSC include dysregulation of wild type p53 (*wt*p53), which is seen in about 96% of HGSC cases. Dysregulation of wtp53 can occur as gain of function (GOF) mutations, termed TP53 mutations. These mutations often lead to the acquisition of oncogenic functions, abrogating the cell cycle constraints controlled by wtp53 [15]. Since wtp53 is a negative transcriptional regulator of RHAMM protein [16] and HGSC datasets such as the CPTAC Data Portal, the Human Protein Atlas and GDC Data Portal revealed few RHAMM mutations in OC specimens, we speculate that dysregulation of wtp53 in OC could drive overexpression of RHAMM. Interestingly, RHAMM was strongly evident in all of the normal FT specimens examined, showing the most intense staining in the distal region of the FT and in nuclei of FTE secretory cells. Similar staining patterns of HGSC and secretory FTE cells align with the current theory of HGSC arising from tubal origins in contrast to LGSC, which is traditionally believed to arise in the OSE.

RHAMM is typically only loosely tethered to the cell membrane [3] suggesting it may be secreted by cells. We showed RHAMM localization to the apical OC cell surface and within cysts of serous OC further suggesting that RHAMM could be secreted. Therefore, we sought to determine whether RHAMM could be detected in bodily fluids. Specifically, we sought to determine whether elevated RHAMM levels could be detected in the urine of OC patients. Urinary markers are ideal in clinical settings since urine collection is simple, safe, non-invasive and cost effective. In addition, urinary proteins retain high stability and urinary filtration precludes the presence of large proteins found in serum, such as albumin, which can confound test results.

In this pilot study, we consistently found elevated urinary RHAMM levels in OC patients that were significantly higher than normal healthy controls and women with benign gynecological disease (*P < 0.0001). While most patients with benign gynecological disease did not demonstrate elevated urinary RHAMM, elevated urinary RHAMM was observed in a patient with uterine fibroids and a patient with endometrioma**.** During inflammation, HA levels increase within the microenvironment which, in turn, promotes increased RHAMM secretion [17] and involvement of RHAMM in the inflammatory process [18]. Consequently, inflammatory benign gynecologic conditions, as may have been present in these two patients, could result in a transient increase in urinary RHAMM levels.

While urinary RHAMM levels were essentially unchanged in patients with benign disease following cytoreductive surgery, reduced urinary RHAMM levels after cytoreductive surgery in OC patients suggests that RHAMM is produced and secreted by OC cells. However, it is important to note that 35.8% of patients experience post-surgical infections following cytoreductive surgery [19] and this may account for increased urinary RHAMM levels in the patients who experienced elevated RHAMM levels at their 2 week follow-up. Nonetheless, utilizing RHAMM for monitoring disease after surgical debulking as a prognostic marker could provide a useful clinical tool for monitoring disease reoccurrence.

RHAMMs’ α-helical and coiled structure confers a hydrophilic and water soluble protein profile [20] providing a mechanism by which RHAMM might be present in bodily fluids. Given that glomerular filtration typically excludes high molecular weight proteins from the urine, we were surprised to detect full length 85 kDa RHAMM protein in OC patient urine by WB. However, our results are in keeping with others who have also reported high molecular weight (MW) proteins in urine of women at high risk for BC including matrix metallopeptidase-2, matrix metallopeptidase-9 (MMP9) and MMP9/neutrophil gelatinase-associated lipocalin complex (MW: 72 kDa, 92 kDa and 115 kDa respectively) [21]. High MW proteins present in the urine are commonly associated with renal dysfunction [22]. Donadio et al. (2003) reported renal impairment in both early and late stage OC showing at least a 10% impairment of glomerular filtration rate and creatinine clearance in 30% of stage 1, 50% of stage 2, 56% of stage 3 and 64% of stage 4 OC patients [22] suggesting that renal impairment due to disease could enable urinary transport of full-length RHAMM. Alternately, despite lacking a secretory signal peptide sequence, RHAMM is commonly secreted from cells by an as yet unknown mechanism [23]. Consequently, RHAMM may bypass glomerular filtration allowing its transport into the urine by a glomerular-independent mechanism.

When analyzing clinical parameters, average urinary RHAMM levels were independent of stage and grade. This is in contrast to our IHC studies where we found an increase in RHAMM by grade and a slight trend for RHAMM to increase by stage. Assmann et al. (2001) showed significantly higher expression of an intracellular splice variant of RHAMM than its cell surface splice variant in BC cells and proposed differential RHAMM splice variant expression for BC pathogenesis [24]. Similarly, the transition from LGSC to HGSC OC may be accompanied by preferential RHAMM splice variant expression so that increased RHAMM staining intensity and cytoplasmic localization noted with increasing grade and stage may reflect a shift away from membranous/extracellular RHAMM production towards increased production of intracellular RHAMM.

Lastly, WB confirmed significantly elevated RHAMM protein in OC cell lysates compared to normal HIOSE cell lines. Therefore, OC cell culture may provide a model system to delineate the molecular and mechanistic function by which RHAMM contributes to OC.

## Conclusion

This pilot study is the first to show RHAMM expression in OC as well as a potential prognostic clinical impact for urinary RHAMM levels. Further, the apparent relationship between increased RHAMM production with increasing tumor grade and stage suggests that RHAMM contributes to OC progression. Lastly, given the shared characteristics of HGSC and FTE reported to date, further studies are clearly warranted to discern the degree to which RHAMM may also be a member of this shared molecular profile.

## Abbreviations

BC: Breast cancer
CD44: Cluster differentiation 44
CRC: Colorectal cancer
ELISA: Enzyme-linked immunosorbent assay
EOC: Epithelial ovarian cancer
FT: Fallopian tube
FTE: Fallopian tube epithelium
GOF: Gain of function
H&E: Hematoxylin and eosin
HA: Hyaluronan
HGSC: High-grade serous cancer
IHC: Immunohistochemical
LGSC: Low-grade serous cancer
LMP: Low malignant potential
MMP9: Matrix metallopeptidase-9
MW: Molecular weight
OC: Ovarian cancer
OSE: Ovarian surface epithelium
RHAMM: Receptor for hyaluronon-mediated motility
RT: Room temperature
TP53: Mutated p53
WB: Western immunoblot
WTP53: Wild type p53
